# Second Annual Meeting of the Southern Dental Association

**Published:** 1870-06

**Authors:** W. N. Morrison


### SELECTED ARTICLES.

### ARTICLE V.

### *Second Annual Meeting of the Southern Deutal Association.*

By W. N. Morrison, D. D. S.

The Convention of the Southern Dental Association commenced its second annual meeting on Wednesday, April 13, at 10 A. M., in the hall of the Mechanics' Institute, Dr. J. G. McAuley, 1st Vice President, in the Chair.

Dr. J. A. Thurber, President of the New Orleans Dental Society, read the welcome address, which was beautifully expressive of the sentiments of the profession in this city to the members of the convention, and was received with marked approbation.

On a call of the roll, by Dr. J. G. Angell, Recording Secretary, the following named gentlemen, active members of the Association, answered to their names:

Drs. W. H. Morgan, Nashville, Tenn.; J. R. Walker, New Orleans; Hy. A. Lawrence, New Orleans; J. G. Angell, New Orleans; G. J. Fredrichs, New Orleans; W.

S. Chandler, New Orleans; J. S. Knapp, New Orleans; W. G. Redmond, Louisville, Ky.

The committee on Membership reported, recommending the following gentlemen for active membership, and they were accordingly elected:

Drs. A. F. McLain, R. M. Lawrence, C. E. Kells, C. L. Thurber, H. Pierson, James West, S. A. Thurber, B. F. Smith, J. A. Casadavant, C. N. Sabourin, Hy. Lawrence, F. Antroux, J. R. Walker, all of New Orleans; W. Johnson, Savannah; E. A. Hermon, Nashville; R. M. Gage, and W. Denson, Mobile; E. Floyd, Fayetteville, N. C.; S. P. Cutler, Holly Springs, Miss.; T. L. Ulmer, Pleasant Hill, Ala.; J. B. Murphy, Atlanta; W. N. Morrison, St. Louis; L. E. Edmonson, Galveston; J. B. Patrick, Charleston; E. R. E. Carpenter, Chicago.

Communications from different members of the Association who were unable to attend, were read, and after some other business of minor interest, the Convention adjourned until 8 o'clock P. M.

*Evening Session.*—Dr. McAuley in the Chair,

The following gentlemen were duly elected permanent members:

T. J. Freidrichs, C. W. Robinson, F. H. Knapp, J. A. De Hart, J. S. Cobb, J. M. Magee, W. N. Morrison, E. R. E. Carpenter.

The Committee on Dental Education, Dr. Gorgas, the chairman, being absent had no report prepared.

Dr. F. Y. Clark read an interesting paper on the subject of appointing dentists in the Army and Navy. The report was received, and approbatory remarks made by Drs. Morgan, Freidrichs, Walker, Magee, Cutler and Cassadavant.

Dr. Ford offered a resolution that the President appoint two delegates to confer with and request the American Dental Association to appoint three members to memorialize Congress on the subject.

On motion of Dr. McLain, the Conventon adjourned until 10 o'clock, A. M. Thursday.

*Thursday*, April 14.—The Convention met pursuant to adjournment; Dr. McAuley in the Chair. Minutes of the previous day were approved.

Drs. G. H. Kirk, of Tuscaloosa, Ala.; T. H. Harrison, Morton, Miss.; were upon ballot elected members.

Dr. Clark read an instructive paper on operative dentistry, and Dr. Morgan one on capping exposed pulps, and on heavy foils.

Dr. Cutler made some remarks on histological conditions and secretions of pus at the root of teeth, and spoke at length and with great interest on microscopical researches.

Dr. Morrison was appointed Corresponding Secretary, and Dr. Thurber, Assistant Secretary.

Dr. Cobb spoke next of the peculiarities of diseased molars, instancing a case of failure, resulting in pyæmic fever in a patient who was but twelve years old. Dr. Cutler expressed the opinion that it was caused by the tunnel-shaped opening at the foramen being imperfectly filled. Interesting remarks were also made on the subject by Drs. Magee, Clark, Walker and Ford.

Dr. McLain thought these ulcerations were induced by arsenious acid, devitalizing the pulp, and that this acid was absorbed to a much greater extent than was supposed.

After other remarks by gentlemen, on a variety of subjects of minor interest, the Convention adjourned till 8 o'clock, P. M.

*Evening Session.*—The President in the Chair.

Charleston, S. C., was selected as the place of the next annual assembly, on the 2d Wednesday of April, 1871.

The Convention adjourned till 10 A. M., Friday.

Previous to the daily sittings of the Convention, we noticed interesting dental operations by Drs. Clark, Carpenter, Shadoan, of Louisville, and others of the Association.

*Friday*, April 15.—The convention met pursuant to adjournment, Dr. Morgan acting President.

Dr. J. S. Knapp read an interesting paper on the subject of wasted alveolar processes and gums, their treatment, etc.' by Dr. W. H. Atkinson, of New York.

The merits of the paper were discussed at length by Drs. Taft, G. J. Freidrichs, J. R. Walker and others.

Dr. Herman, of Nashville, mentioned a difficult case of alveolar abscess, in which he had used the usual remedies unsuccessfully.

Dr. J. S. Knapp, advised the use of creosote in alternation with chloride of zinc.

Dr. Clarke, of Savannah, recommended carbolic acid, iodine and glycerine.

Meeting adjourned at half-past two, P. M., to attend the dinner given by the New Orleans Dentists, to the members of the Association, at the Washington Hotel, Lake Pontchartrain. Representatives of the press, and quite a number of ladies were present.

The tables were handsomely decorated with flowers and each guest was presented with a bouquet. Wines of the finest brands in abundance. Strawberries, bananas and other native and tropical fruits were especially enjoyed by the assembled company.

The affair passed off most entertainingly. A number of toasts were given, to which appropriate responses were made by Dr. F. H. Knapp, and Dr. J. A. Thurber, who respectively occupied the ends of the table. Drs. Taft, Casadavant, Walker, Morgan, and others of the profession, were called out and made very pleasant little speeches. Also, representatives from the medical profession and the press, responded to their calls.

One of the most pleasant features of the occasion was the presentation of a elegant gold-headed cane, by the Association, as a mark of esteem, to Mr. Samuel Hape, of Atlanta, Ga.—Dr. Morgan making the presentation speech.

*Evening Session.*—On re-assembling, at eight o'clock, as per adjournment, the members all felt in such good humor that Dr. Patrick called for Dr. Cutler to sing, "Shoo Fly," with the Association to join in the chorus.

Dr. Angel introduced a resolution that the Association take into consideration the importance of establishing a Dental Journal, South; which was adopted.

Dr. Angel, being called upon, then expressed his views, as chairman of a committee from the New Orleans Dental Association, of the importance of having an independent journal established.

Dr. Morrison of the "Missouri Dental Journal," and Dr. Taft of the "Dental Register," being called upon, gave their views upon the subject. They fully concurred in the opinion that there was room for another journal. Other members entertained the same views, and a committee of five, consisting of Drs. J. S. Knapp, (La.;) A. C. Ford, (Ga.;) J. B. Patrick, (S. C.;) W. H. Shadoan, (Ky.;) and R. M. Gage, (of Ala.) was appointed to take it into consideration and report at the 10 A. M. session on to-morrow.

Dr. Cobb asked if it was possible for bad teeth in any way to produce cancer.

He was answered negatively by Drs. Clarke, F. H. Knapp, J. R. Walker, McLain and Magee.

Dr. Cutler was of the opinion that they might do so.

Dr. B. F. Smith read an excellent essay on the "Past and Present of Dentistry."

On motion of Dr. Walker, the meeting adjourned until 10 A. M. to-morrow, when the subject of Mechanical Dentistry was to be taken up.

The clinic operations this morning were of a very interesting nature. Dr. J. N. Knapp, operating in the mouth of Dr. McAuley, the President of the Association, made, in the handsomest and most skillful manner, a compound filling, with cylinders. The operation, and the manufacture of the cylinders by Dr. Knapp, were watched with great interest by the assembled members of the profession.

Dr. Shadoan, of Louisville, also made a handsome front filling for Dr. Clark, of Savannah.

*Saturday Morning.*—Association called to order by Dr. McAuley.

On motion of Dr. Cutler, reading of the minutes was dispensed with, and the subject of Mechanical Dentistry was called by Dr. Walker, who thought rubber work had been a curse to the profession.

Dr. Taft thought mechanical dentistry should be separated from the operative entirely.

Dr. Cutler wanted a higher style of mechanical dentistry under the direction of the operative dentist. After remarks from others the subject was closed and miscellaneous business taken up. It was ordered that three hundred copies of the Constitution, By-laws, and Proceedings of the Association be printed, and one hundred be bound for immediate distribution.

At twelve o'clock the election of officers for the ensuing year resulted as follows:

President—Dr. James S. Knapp; 1st Vice President—Dr. F. Y. Clark; 2d Vice President—Dr. E. A. Herman; 3d Vice President—Dr. L. E. Edmonson; Corresponding Secretary—Dr. W. N. Morrison; Recording Secretary—Dr. John G. Angell; Treasurer—Dr. W. G. Redmond; Executive Committee—Drs. J. B. Patrick; G. Taff; A. M. Ford; W. S. Chandler, and Samuel Rambo.

The following named gentlemen were also elected Delegates to the American Dental Association, which meets in Nashville on the first Tuesday in August: R. N. Lawrence; —— Holmes; J. R. Walker; A. F. McLain; J. B. Patrick; R. M. Gage; E. Floyd; W. H. Burr; S. G. Holland; H. A. Lawrence; F. Anfoux; Louis Augspath; B. F. Smith; J. B. Murphy; L. E. Edmonson.

After a vote of thanks to the members of the press, hotels, railroads, steamers, and retiring officers, the Association adjourned to meet in Charleston, S. C., on the second Monday in April, 1871.

